# Quantization and diagnosis of *Shanghuo (Heatiness)* in Chinese medicine using a diagnostic scoring scheme and salivary biochemical parameters

**DOI:** 10.1186/1749-8546-9-2

**Published:** 2014-01-04

**Authors:** Sijun Liu, Zhaosheng Huang, Qingguang Wu, Zhangjie Huang, Lirong Wu, Wenli Yan, David Lungpao Chang, Zheng Yang, Zongwei Wang

**Affiliations:** 1 Guangzhou University of Traditional Chinese Medicine, 12 Jichang Road, Guangzhou 510405, China; 2 Colgate-Palmolive (China) Co. Ltd., Guangzhou, China; 3 Massachusetts General Hospital, Harvard Medical School, 55 Fruit St., Warren 317, Boston, MA 02114, USA

## Abstract

**Background:**

This study aims to establish a diagnostic scoring scheme for *Shanghuo (Heatiness)* and to evaluate whether *Shanghuo* is associated with biochemical parameters of salivary lysozyme (LYZ), salivary secreted immunoglobulin (S-IgA), salivary amylase (AMS), and saliva flow rate (SFR).

**Methods:**

We collected 121 *Shanghuo* patients at the Affiliated Hospitals of Guangzhou University of Traditional Chinese Medicine in Guangdong Province, 60 cases as a *Shanghuo* recovered group, and 60 healthy cases as a healthy control group. The diagnostic scoring scheme was established by probability theory and maximum likelihood discriminatory analysis on the basis of epidemiology with the design of self-controlled clinical trial. Subsequently, we used the same methods to collect 120 *Shanghuo* patients, 60 *Shanghuo* recovered cases, and 60 healthy cases in both Hunan Province and Henan Province. The levels of LYZ, S-IgA, AMS, and SFR were tested when the patients suffered from *Shanghuo* or recovered, respectively.

**Results:**

The diagnostic score table for *Shanghuo* syndrome was established first. In the retrospective tests, the sensitivity, specificity, accuracy, and positive likelihood ratio of the diagnostic score table were 98.9%, 93.5%, 97.5%, and 14.34%, respectively. In the prospective tests, the corresponding values were 94.9%, 85.7%, 91.7%, and 6.64%, respectively. *Shanghuo* was classified into three degrees based on the diagnostic scores, common *Shanghuo*: 63–120; serious *Shanghuo*: 121–150; very serious *Shanghuo*: >150. A negative correlation was found between *Shanghuo* and S-IgA (*R* = -0.428; *P* = 0.000). The level of S-IgA was also affected by seasonal and regional factors. No significant correlations were found between *Shanghuo* and the levels of LYZ, AMS, and SFR.

**Conclusions:**

In this study, *Shanghuo* could be diagnosed by the combination of the diagnostic score table and S-lgA level.

## Background

*Huo* (fire) is one of the five basic elements in traditional Chinese Medicine (TCM), together with *Mu* (wood), *Tu* (earth), *Jin* (metal), and *Shui* (water). *Huo* symbolizes active, positive, and warm substances or states [1]. When *Huo* becomes hyperactive in the body, this kind of internal *Huo* or internal *Re* (heat) is idiomatically expressed as “*Shanghuo*” (*Heatiness*) [2].

*Shanghuo* in traditional Chinese medicine (TCM) is characterized by “redness, swelling, fever, and pain”, usually emerges in the middle to late stages of infectious diseases, and also occurs in non-infectious diseases such as autoimmune diseases and endocrine diseases [3]. Typically, TCM physicians diagnose *Shanghuo* through inspection (visual examination), audio-olfactory examination, inquiry, and palpation, which are usually based on the clinicians’ subjective judgment, not quantified objective diagnostic parameters.

Human saliva is composed of 99.5% water, and the remaining 0.5% comprises electrolytes, mucus, glycoproteins, enzymes, and antibacterial compounds such as secretory IgA (S-IgA) and lysozyme (LYZ) [4-6]. S-IgA and LYZ protect the tooth enamel and prevent tooth decay and gum disease [7,8]. Studies have shown that some of the components in saliva can be used as biomarkers for diagnosis. Real-time PCR assays of both liquid and dried saliva specimens showed high sensitivity and specificity for detecting cytomegalovirus infection, suggesting their potential as screening tools for cytomegalovirus infection in newborns [9]. Zerr *et al.*[10] developed a noninvasive method for testing serially-collected saliva specimens for human herpesvirus-6, and applied this method prospectively in children from birth to 2 years of age to determine the pattern of acquisition and natural history of human herpesvirus-6 infection. More importantly, the easy access to saliva samples provides superiority for good repeatability and improved diagnostic accuracy.

In the oral immune system, S-IgA has been recognized as a critical immune barrier for infection and allergy [11]. Studies have shown that S-IgA agglutinates and binds the microbes in the oral cavity, inhibits their adhesive capability, and prevents them from clinging to the surface of the oral tissues. In addition, S-IgA inhibits bacterial growth and proliferation by changing the growth features of bacteria, and inhibits viral duplication with the help of complement proteins and lysosomes. Studies have found that the levels of S-IgA are evidently lower in patients with recurrent aphthae, recalcitrant oral ulceration, and oral lichen planus than in healthy control subjects [12-15].

Previous studies suggested that LYZ and SFR are associated with the occurrence and development of common oral diseases [16,17]. In general, a low level of LYZ is often correlated with the occurrence of oral infections and inflammatory diseases, and the frequency and proneness for dental plaque and dental caries [16].

This study aims to include the biochemical parameters of salivary LYZ, salivary S-IgA, salivary amylase (AMS), and saliva flow rate (SFR) in the diagnosis of the clinical syndrome of *Shanghuo*. We investigated *Shanghuo* cases from Guangdong, Hunan, and Henan provinces in China by establishing a diagnostic score table and a frequency table for *Shanghuo*, and evaluated whether *Shanghuo* is associated with the levels of LYZ, AMS, SFR, and S-IgA.

## Methods

This study followed the Standards for Reporting of Diagnostic Accuracy (STARD) [18]. And it was approved by the Ethical review board of The First Affiliated Hospital of Guangzhou University of Chinese Medicine, Ethical Committee of Hunan University of TCM, and Ethical Committee of Henan University of TCM. All candidates signed a written consent followed by a baseline interview. During the interview, the candidates were asked to fill out a questionnaire, and the collected data included candidate’s name, age, gender, address, the degree situation of observed index, the collection location, the collection time and the diagnostic results.

### Inclusion criteria

#### *Shanghuo* group

The subjects in the *Shanghuo* group, who signed a written consent that approved by the research ethical committee complied with the diagnostic standards described below [19] and were simultaneously diagnosed as suffering from *Shanghuo* by three independent doctors in our group. If there was any disagreement on the diagnostic results among the doctors, the subject was excluded. The standards were as follows: internal exuberant *fire* and *heat*, characterized by common symptoms such as fever, thirst, and preference for cold drinks, epigastric burning, flushed face and hot eyes, constipation, scanty yellow urine, reddened tongue with yellow and dry coating, rapid pulse or vigorous and rapid pulse.

#### *Shanghuo* recovered group

The subjects in the *Shanghuo* recovered group were diagnosed as suffering from *Shanghuo* at the beginning of the study and recovered after 1–2 weeks. These subjects were also identified by three independent doctors in our project group with complete agreement. The recovered criteria matched those applied to the healthy control group.

#### Healthy control group

The subjects in the healthy control group had no excess *Huo* (or *Re*) syndrome, *Xu Re* (deficiency *heat*) syndrome, *Xu Huo* (deficiency *fire*) syndrome, for instance, oral dryness, oral ulcers, bitter taste, halitosis, swollen gums with pain or bleeding, stuffy nose, nasal dryness, nosebleed, ocular itch, eye gum, tinnitus, throat dryness, sore throat, acne, vertigo, scurf, vexing heat, low fever, insomnia, irascibility, yellow urine, reddened tongue, yellow tongue coating, constipation, thin rapid pulse, rapid flooding pulse, and were simultaneously diagnosed as not suffering from *Shanghuo* by three independent doctors in our group.

### Exclusion criteria

The exclusion criteria for three groups were as follows: patients with malignant tumor diseases, patients with hepatitis, tuberculosis or other infectious diseases, patients with mental disorders, patients with diseases affecting the procedure for collecting saliva or affecting the follow-up study, non-acceptance of providing informed consent or low compliance from the patient.

### Collection of clinical data

**Figure 1:**
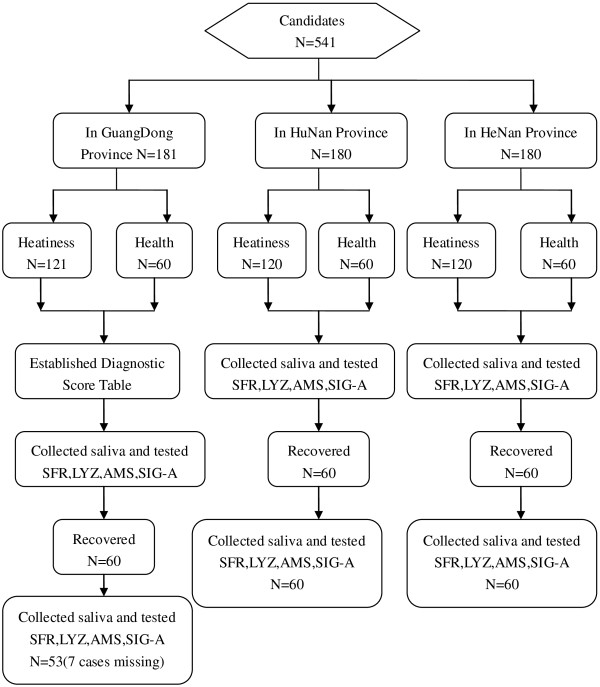
The flow diagram of the study.

Between June 2003 and June 2008, we collected 121 *Shanghuo* patients at the Affiliated Hospitals of Guangzhou University of Traditional Chinese Medicine in Guangdong Province, 60 cases as the *Shanghuo* recovered group, and 60 healthy control cases as well––– to establish the diagnostic score table for *Shanghuo*. The same standards were followed when we collected another 120 *Shanghuo* cases, 60 recovered cases, and 60 healthy cases from both Hunan Province and Henan Province (Table 1). As showed in Table 1A, there were 102 male and 139 female in Guangdong province, 113 male and 127 female in Hunan province, 105 male and 135 female in Henan province. By chi-square test, there was no significant difference between three geographical regions in gender (*P* > 0.05, X^2^ =1.162, *P* = 0.559). Through the test of homogeneity of variance, age and weight of subjects in three geographical regions has homogeneity of variance. By One-way ANOVA test (LSD), no significant difference was found between two of three groups in the factor of age and weight (*P* = 0.126, 0.197 and 0.144 respectively, with the age of subjects in Guangdong *vs*. Hunan, Guangdong *vs*. Henan, and Hunan *vs*. Henan. *P = *0.082, 0.095, and 0.104 respectively, with the weight of subjects in Guangdong *vs*. Hunan, Guangdong *vs*. Henan, and Hunan *vs*. Henan Table 1B). Patient information was further summarized as Figure 1.

**Table 1:** The clinical data of subjects

**A. The number of subjects from three geographical regions**										
**Gender**	**Number of subjects in Guangdong province**			**Number of subjects in Hunan province**			**Number of subjects in Henan province**			**Total**
	**Heatiness**	**Recovered**	**Healthy control**	**Heatiness**	**Recovered**	**Healthy control**	**Heatiness**	**Recovered**	**Healthy control**	
Male	51	24	27	55	28	30	50	30	25	318
Female	70	36	33	65	32	30	70	30	35	391
Total	121	60	60	120	60	60	120	60	60	721
**B. The situation of age and weight with subjects**										
**Geographical regions**				**Number of subjects (N)**			**Age (year)**		**Weight (kg.)**	
Subjects in Guangdong province				241			32.415 (15.220 )		49.541 (17.040)	
Subjects in Hunan province				240			33.250 (19.085)		52.440 (15.115)	
Subjects in Henan province				240			31.215 (12.550)		54.755 (13.205)	

All data were presented as mean (SD).

The patients in the three groups were asked to fill out a questionnaire (Additional file 1). This questionnaire on symptoms and signs was established by comprehensively listing the frequency of occurrence of symptoms and signs of *Shanghuo* according to the literature, selecting the symptoms with their frequency of occurrence with ranking of top 30 as the indices for four diagnostic methods, and setting a dichotomous variable (Yes/No).

### Saliva collection and salivary biochemical parameters test

#### Sampling procedure

Saliva samples were collected from 9:00–11:00 a.m. and 2:00–4:00 p.m., respectively. After rinsing the mouth with clean water, the subjects sat quietly for 3 minutes keeping the saliva in their mouth naturally, and then spat the saliva into a vial (non-irritant saliva) per minute. Saliva was collected for 10 minutes and the SFR was calculated (shown in 4.2.1). If the total amount of saliva over 10 minutes was less than 3 mL, the collection duration was prolonged until 3 mL of saliva had been collected, and the total time was recorded for calculation of the SFR. The collected samples were stored at -20°C until analysis.

### Determination of SFR, AMS, LYZ and S-IgA

#### SFR

The SFR (mL/min) was calculated as the total volume of saliva collected per collecting time (total volume of saliva divided by minutes required).

#### AMS

After freeze-thawing, the saliva samples were diluted 20 times in saline. The level of AMS was determined by a commercially available AMS kit (Salivary Amylase Kit; HUMAN GmbH Co., Germany; Catalogue No. H100) in accordance with the manufacturer’s protocol.

#### LYZ

The level of LYZ was examined using LYZ kits (Nanjing Jiancheng Bioengineering Research Institute, China; Catalogue No. 20060922, 20061025, and 20070412) in accordance with the producer’s guidelines, by a 752 Spectrophotometer (Shanghai Jingke Equipment Co., Ltd., China).

#### S-IgA

S-IgA was determined with S-IgA Kits (Beijing North Institute of Biotechnology, China; Catalogue No. 061120, 061230, 070412, and 070420) by an SN-695B radioimmunoassay measuring instrument (Shanghai Hesuo Rihuan Photoelectric Instrument Co., Ltd., China).

### Statistical analysis

The chi-square test was used to analyze the observed indices. A method for maximum likelihood discriminatory analysis was used to establish the diagnostic score table for *Shanghuo*[20]. The Wilcoxon rank sum correlation test was used to analyze the correlations between the *Shanghuo* scores and the levels of SFR, LYZ, AMS, or S-IgA. A paired sample *t*-test was used to study the seasonal and regional differences in S-IgA between the *Shanghuo* patients and healthy controls. All of the statistical tests were two-tailed. Values of *P* < 0.05 were considered as statistical significance.

## Results

### Diagnostic score tables

We calculated the number of *Shanghuo* cases and healthy cases with the occurrence of each symptom index among the 30 indices on a one-by-one basis with the chi-square test, and excluded the indices with values of *P* > 0.05.

Healthy negative means that the number of the index did not appear in the healthy control group, while healthy positive means the number of the index did appear in the group. Similarly, *Shanghuo* negative means that the number of the index did not appear in the *Shanghuo* group, while *Shanghuo* positive means the number of the index did appear in the group. Using the chi-square test, we found that (5) Constant hunger, (12) Red eyes, and (17) Sputum showed no significant differences between the healthy control and *Shanghuo* groups (*P* = 0.659 for constant hunger, *P* = 0.150 for red eyes and *P* = 0.117 for Sputum). However, the remaining 27 indices did differ significantly between the healthy control and *Shanghuo* groups ( *P* = 0.004 for Stuffy nose, *P* = 0.012 for Nosebleed, *P* = 0.004 for Scurf, *P* = 0.003 for Reddened tongue, *P* = 0.002 for Rapid flooding pulse, and *P* = 0.000 for the other 22 indices). Therefore, these 27 indices were retained (Table 2).

**Table 2:** The result of chi-square test

**Indices**	**Healthy negative**	**Healthy positive**	**Heatiness negative**	**Heatiness positive**	**Chi-square value**	***P***
(1)Ulcer	60	0	39	82	74.340	0.000
(2)Oral dryness	50	10	20	101	75.475	0.000
(3)Bitter taste	59	1	84	37	20.214	0.000
(4)Halitosis	60	0	81	40	25.462	0.000
(5)Constant hungering	45	15	87	34	0.195	0.659
(6)Swollen gums with pain or bleeding	55	5	23	98	86.349	0.000
(7)Stuffy nose	55	5	89	32	8.092	0.004
(8)Nasal dryness	58	2	57	64	42.523	0.000
(9)Nosebleed	60	0	109	12	6.373	0.012
(10)Ocular dryness	50	10	46	75	33.071	0.000
(11)Ocular itch	57	3	87	34	13.160	0.000
(12)Red eyes	59	1	113	8	2.076	0.150
(13)Eye gum	59	1	66	55	35.994	0.000
(14)Tinnitus	59	1	101	20	20.984	0.000
(15)Throat dryness	57	3	56	65	40.591	0.000
(16)Sore throat	59	1	83	38	20.984	0.000
(17)Sputum	57	3	106	15	2.450	0.117
(18)Acne	48	12	55	66	19.519	0.000
(19)Vertigo	55	5	90	31	7.522	0.006
(20)Scurf	55	5	89	32	8.092	0.004
(21)Vexing heat	60	0	83	38	23.85	0.000
(22)Low fever	60	0	89	32	19.276	0.000
(23)Insomnia	53	7	57	64	28.596	0.000
(24)Irascibility	59	1	67	54	34.998	0.000
(25)Yellow urine	55	5	72	49	19.821	0.000
(26)Constipation	53	7	61	60	24.739	0.000
(27)Reddened tongue	58	2	97	24	8.879	0.003
(28)Yellow tongue coating	59	1	83	38	20.984	0.000
(29)Thin rapid pulse	55	5	62	59	28.681	0.000
(30)Rapid flooding pulse	60	0	104	17	9.304	0.002

Next, we calculated the exponential value of each index in the healthy control and *Shanghuo* groups by the conditional probability conversion formula [20]. The formula was:

$$\begin{array}{l} L_{\mathrm{ij}}=\left[\mathrm{lg}\left(X_{j}/Y_{i}\right)+1\right]\times 10\left(X_{j}/Y_{i}>0\right)\mathrm{and}\;L_{\mathrm{ij}}\\ \;=-10\left(X_{j}/Y_{i}-0\right), \end{array}$$

where L_ij_ is the exponential value of the index, X_j_ is the number of *Shanghuo* or healthy cases with the index, and Y_i_ is the total number of cases collected.

By using the frequency of occurrence of each index, the exponential value of each index in the healthy cases was subtracted and the absolute value was adopted. Similarly, the exponential value of each index in the *Shanghuo* cases was subtracted and the absolute value was adopted. Subsequently, the two absolute values were summed up to calculate the score of each index. For example, for all 60 cases in the healthy group, (1) Ulcer did not appear. Thus, the negative conditional probability of ulcer was 100% and the positive conditional probability of ulcer was 0%. According to the conditional probability conversion formula, the negative exponential value of ulcer in the healthy group was 10 and the positive exponential value was -10. The negative exponential value of ulcer in the *Shanghuo* group was 5 and the positive exponential value was 8. Therefore, the final score for ulcer was |10 - (-10)| + |5 - 8| = 23 (Additional file 2).

After simplification of the above table, we obtained a diagnostic score table for *Shanghuo* (Table 3).

**Table 3:** **Diagnostic score table of *heatiness***

**Indices**	**Value**	**Indices**	**Value**	**Indices**	**Value**
(1)Ulcer	23	(10)Eye itching	8	(19)Low-grade fever	15
(2)Dry mouth	14	(11)Secretion of the eyes	18	(20)Insomnia	8
(3)Bitterness in the mouth	15	(12)Tinnitus	11	(21)Tantrum	17
(4)Bad breath	17	(13)Pharyngoxerosis	13	(22)Yellow urine	9
(5)Gum swelling and aching or bleeding	17	(14)Sore throat	15	(23)Constipation	8
(6)Nasal obstruction	6	(15)Acne	6	(24)Red tongue	9
(7)Nasal dryness	15	(16)Dizziness	6	(25)Yellow coating	15
(8)Nose bleeding	10	(17)Scurf desquamation	6	(26)Frequent and weak pulse	11
(9)Dryness of the eyes	9	(18)Dryness-heat	17	(27)Frequent and strong pulse	13

### Diagnostic threshold values

At the beginning of diagnosis, each index in Table 2 was assumed “negative”, and the exponential values of the 27 “negative” indices of *Shanghuo* in Table 3 were summed to obtain the exponential value sum for *Shanghuo*, *i.e.*, 202. The exponential value sum for non-*Shanghuo* obtained in a similar way was 265, and the relative exponential sum of value for *Shanghuo* was 63. In other words, greater than 63 of the total score for “positive” indices is the diagnostic threshold value for *Shanghuo*.

### Clinical application

**Figure 2:**
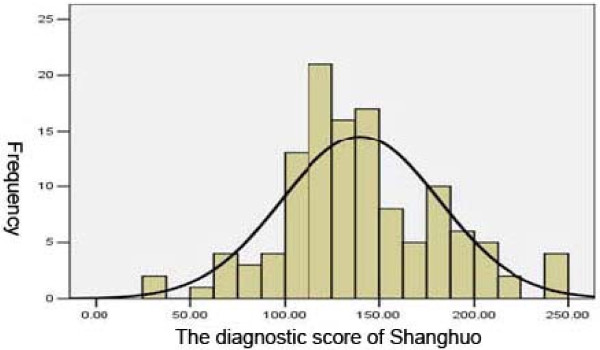
The normal distribution test.

The test for normality of the total cases collected in Guangzhou, Henan, and Hunan provinces in reference to the diagnostic score table showed that the score values for severity of the *Shanghuo* cases were consistent with a normal distribution (Figure 2). Statistically, if a digital index is consistent with a normal distribution, the percentage grading will be applicable.

According to the method for percentiles (*p*): *P*_33.3_ =120.63 ≈ 120; *P*_66.7_ =149.37 ≈ 150, *Shanghuo* could be classified into three degrees based on the diagnostic scores: common *Shanghuo*: 63–120; serious *Shanghuo*: 121–150; very serious *Shanghuo*: > 150.

### Diagnostic tests

#### Retrospective tests

By virtue of the established diagnostic score table for *Shanghuo*, we first diagnosed the 361 cases (including all *Shanghuo* cases in the three regions) in conformity with the *Shanghuo* diagnostic standards, and calculated that the sensitivity, specificity, accuracy, and positive likelihood ratio were 98.9%, 93.5%, 97.5%, and 14.34%, respectively.

### Prospective tests

We diagnosed the 180 cases (including all healthy cases in the three regions) by virtue of the established diagnostic score table for *Shanghuo*, and found that the sensitivity, specificity, accuracy, and positive likelihood ratio of the test were 94.9%, 85.7%, 91.7%, and 6.64%, respectively.

### Assessment of diagnostic efficiency with a ROC curve

**Figure 3:**
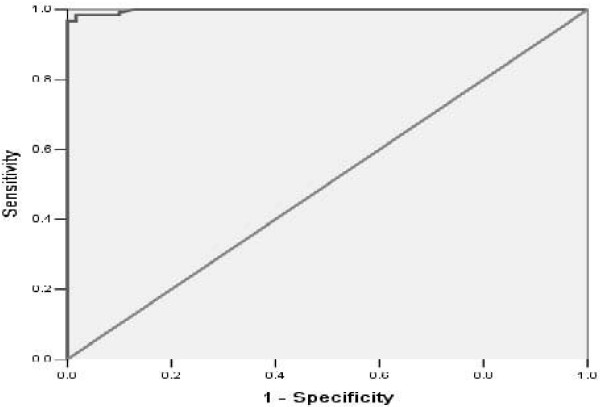
Assessment of diagnostic efficiency with a ROC curve.

We assessed the cases collected from the three geographical regions by the diagnostic score table. Taking the sensitivity as the ordinate axis and the specificity as the abscissa axis, we obtained a receiver operating characteristic (ROC) curve to appraise the diagnostic efficiency by calculating the area under the ROC curve (Figure 3). The size of the integrated area under the curve was closely related to the reliability of a diagnostic trial.

### Analysis of comparisons between *Shanghuo* and S-IgA

#### Comparisons between the *Shanghuo* group and *Shanghuo* recovered group for salivary biochemical parameters

The SFR values were 0.430 ± 0.290 mL/min and 0.450 ± 0.230 mL/min before and after *Shanghuo* recovery, respectively (*P* = 0.503). The levels of LYZ and AMS did not show any significant differences before and after *Shanghuo* recovery (*P* = 0.300 and *P* = 0.240, respectively). However, the level of S-IGA was significantly elevated from 439.770 ± 60.710 mg/dL with *Shanghuo* to 1348.570 ± 170.690 mg/dL after recovery (*P* < 0.001) (Table 4).

**Table 4:** Self-paired comparison of the parameter changes

**Index**	**Status:**	**N**	**Value**	**T**	***P***
SFR	*Heatiness*	60	0.430 (0.290)	-0.673	0.503
	Recovered	60	0.450 (0.230)		
LYZ	*Heatiness*	60	33.420 (5.830)	3.087	0.003**
	Recovered	60	34.250 (5.200)		
AMS	*Heatiness*	60	112916.000 (83073.000)	2.315	0.024*
	Recovered	60	87309.000 (86224.000)		
S-IgA	*Heatiness*	60	439.770 (607.100)	-4.530	0.000***
	Recovered	60	1348.570 (1706.900)		

All data were presented as mean (SD). *P < 0.05, **P < 0.01, ***P < 0.001, by paired *t* test.

### Correlations between *Shanghuo* and the levels of SFR, LYZ, AMS, and S-IgA

The statistical analyses of the *Shanghuo* scores and the correlations between *Shanghuo* and the levels of SFR, LYZ, AMS, and S-IgA were performed by the Wilcoxon rank sum correlation test, using the corresponding index of the same patient. The data suggested that there was a significantly negative correlation between the diagnostic score for *Shanghuo* and S-IgA (*R* = -0.428; *P* = 0.000), but no significant correlation between the other indices and the diagnostic score for *Shanghuo* (Table 5).

**Table 5:** **Correlation of the diagnostic score of *Shanghuo *** **with the saliva biochemical parameters**

	**N**	**P**	**R**
Score and SFR	174	0.973	-0.627
Score and LYZ	174	0.592	0.224
Score and AMS	174	0.121	0.365
Score and S-IgA	174	0.000***	-0.428

Data from Guangzhou province, included 121 *heatiness* cases and 60 healthy cases, excluded 7 cases whose saliva biochemical parameters were not available. ***P < 0.001, by paired *t* test.

### Factors associated with S-IgA acquisition

To examine whether S-IgA had seasonal effects on *Shanghuo*, we collected and evaluated 361 *Shanghuo* cases collected from above three regions (121 cases in Guangdong Province, 120 cases in Hunan Province, and 120 cases in Henan Province) for seasonal divisions on the basis of the 24 solar terms in the lunar calendar (Table 6). The results demonstrated that there were significant differences between spring and summer (*P* = 0.000), spring and autumn (*P* = 0.000), spring and winter (*P* = 0.000), summer and winter (*P* = 0.047), summer and autumn (*P* = 0.000), and autumn and winter (*P* = 0.000) (Table 6A). The different levels of S-IgA in different seasons suggested that the seasonal factor might be considered when studying *Shanghuo* and S-IgA.

**Table 6:** Seasonal and regional comparison of S-IgA

**A: Seasonal comparison of S-IgA**		
**Season**	**N**	**Value of S-IgA**
Spring	89	647.177 (159.324)*
Summer	92	478.598 (138.558)*
Autumn	84	861.726 (75.147)*
Winter	96	513.573 (86.463)*
**B: Regional comparison of S-IgA**		
**Regions**	**N**	**Value of S-IgA**
Guangdong	121	604.891 (171.961)^#^
Hunan	120	720.917 (207.122)
Henan	120	697.73 (188.096)

All data were presented as mean (SD). *P< 0.01, *vs*. any other seasons; ^#^*P* < 0.01, *vs*. any other provinces.

Through comparisons among groups of cases from different regions, we found that there were significant differences between Guangzhou and Henan (*P* = 0.000) and Guangzhou and Hunan (*P* = 0.000), but no significant difference between Hunan and Henan (*P* = 0.116) (Table 6B). These data suggested that the levels of S-IgA might be affected by region.

### Evaluation of *Shanghuo* by combination of the diagnostic score tables and S-IgA levels

Finally, we investigated the consistency of *Shanghuo* cases collected from Guangzhou, Henan, and Hunan provinces before and after the experiment, using the S-IgA level and diagnostic score table from Guangzhou. After the clinical diagnosis was executed by three clinicians individually, the D values were compared with the diagnostic score table, and the S-IgA levels without a quantitative threshold value were compared with the trend. The results showed that for diagnosis with the D value independently, the consistency with the clinical diagnosis of *Shanghuo* was 89.6%. When evaluating S-IgA independently, the consistency with the clinical diagnosis of *Shanghuo* was 48.2%. For combined evaluation with D value and S-IgA, the consistency with the clinical diagnosis of *Shanghuo* was 92.2%. These data indicated that evaluation by combining D value and S-IgA increased the diagnostic efficiency for *Shanghuo*.

## Discussion

As one essential element in TCM, *huo* provides energy and power to human body. *Shanghuo* is a state that *huo* becomes hyperactive and aggressive. The oral ailments associated with *Shanghuo* syndrome include oral dryness, mucosal ulcer, and gum bleeding, swelling, and pain. These symptoms are similar to those of Sjogren syndrome, recurrent aphthae, gingivitis, periodontitis, and caries [21].

The production of saliva is affected by many factors, such as temperature, food, mood, and different times of the day [4]. To make saliva samples more repeatable, we developed a self-controlled experiment and collected saliva in the same time windows, *i.e.*, 9:00–11:00 a.m. and 2:00–4:00 p.m.

In this study, we found that the level of S-IgA was significantly decreased in the *Shanghuo* group compared with the healthy control group, suggesting that S-IgA might represent a diagnosic marker for *Shanghuo*. Further analyses indicated that the level of S-IgA was negatively correlated with the severity of *Shanghuo*. The levels of S-IgA in *Shanghuo* patients varied among different seasons and regions, suggesting that the influences of seasons and regions should be considered when S-IgA was used as a marker for *Shanghuo*.

According to our clinical observations, many *Shanghuo* patients had xerostomia, albeit to different extents. As the first study on the correlation between xerostomia, SFR, and *Shanghuo*, we investigated whether the SFR in patients was associated with *Shanghuo*. However, our data did not show any direct evidence for this hypothesis. Further studies need to be undertaken to address this issue.

Quantitative diagnosis is an interdisciplinary subject and is the probability theory of statistics for the treatment effects evaluation and prognostic diagnosis [22]. *Shanghuo* involves many viscera, with different manifestations in different viscera, and there have been no clear definitions for the essence and diagnostic standards of *Shanghuo*. Here we established a diagnostic scoring scheme with the methods of probability theory and maximum likelihood discriminatory analysis on the basis of epidemiology.

From the diagnostic score table and S-IgA before and after *Shanghuo* and the consistency with the clinical diagnosis, we found that the combination of the diagnostic score table and S-IgA could increase the diagnostic efficiency. The diagnostic score table is intuitive and capable of direct diagnosis, albeit through 27 diagnostic indices of TCM, if tongue coating and pulse are diagnosed by modern machines [23]. We established the diagnostic score table by statistical methods and made an attempt for the diagnosis of *Shanghuo* quantitative research. In addition, we used modern machines for tongue coating and pulse, applied the diagnostic score table, and found that the scores for patients with *Shanghuo* could be calculated to indicate the severity of the *Shanghuo,* and that these scores could be used to evaluate the effectiveness of curative treatments.

In this study, we found that the levels of LYZ, AMS, and SFR had no significant correlations with *Shanghuo*, while S-IgA was associated with *Shanghuo* even the S-lgA level might be varied by different regions and seasons. We believe that quantification using the diagnostic score table and the level of S-IgA will improve the diagnostic accuracy and efficiency of *Shanghuo*.

Still, some limitations of this work should be considered. First, participants were not randomly selected and were limited to the three particular geographic regions in China. And the study was generally characterized by small sample sizes and may not be representative of the population. Secondly, the collection of data was objective because of lacking gold standard for *Shanghuo*, even though the recruited candidates simultaneously diagnosed by three independent doctors. These design and sampling strategies may reduce the external validity of our study. Also, because of the situation of pulse was included in the diagnostic score table, when this diagnostic scoring scheme was applied in clinic, the theory of pulse in TCM should be known. These limitations need to be considered when our findings are interpreted.

## Conclusions

This study demonstrated that *Shanghuo* could be diagnosed by a combination of diagnostic score tables and S-lgA levels.

## Abbreviations

LYZ: Salivary lysozyme; S-IgA: Salivary secreted immunoglobulin; AMS: Salivary amylase; SFR: Saliva flow rate; TCM: Traditional Chinese Medicine.

## Competing interests

The authors declare that they have no competing interests.

## Authors’ contributions

ZH, SL, QW, DC, ZY and ZW conceived and designed the study. SL performed the experiment. SL, ZH, LW and WY performed the statistical analysis. SL and ZW wrote the manuscript. All authors read and approved the final manuscript.

## Supplementary Material

Additional file 1 **The representative questionnaire used for *heatiness *** **and control subjects.** Click here for file

Additional file 2 **The indexs and values of *heatiness *** **symptom.** Click here for file
