# Supplementary material for: Quantization and diagnosis of Shanghuo (Heatiness) in Chinese medicine using a diagnostic scoring scheme and salivary biochemical parameters
Source: Chin Med. 2014 Jan 4;9:2. doi: 10.1186/1749-8546-9-2 (PMC3891990; doi:10.1186/1749-8546-9-2)
Supplement: Additional file 1 — The representative questionnaire used for heatiness and control subjects. [file 1749-8546-9-2-S1.doc]

**Questionnaire on candidates**

The investigation is organized by GuangZhou University of TCM with the purpose of evaluating the effectiveness of *heatiness*. This questionnaire will be completed by yourself under the researcher’s guidance. The tongue coating and the pulse condition will be also recorded on the questionnaire by the researcher. The data presented as an average of the results or individual results will be shown without identifying you by name as a subject. Thank you!

NO.：

1. Basic Information：

Name： Gender： Age： Weight(kg.) ： Profession： Native place： Address：

Tel.： E-mail：

2. Do you have ulcer in month? NO （0） YES （1）

3. Do you feel dry in mouth? NO （0） YES （1）

4. Do you feel bitter taste of mouth？ NO （0） YES （1）

5. Do you have halitosis? NO （0） YES （1）

6. Do you have uloncus？ NO （0） YES （1）

7. Do you have nasal obstruction？ NO （0） YES （1）

8. Do you feel dry in the nasal cavity？ NO （0） YES （1）

9. Do you have nose bleeding ？ NO （0） YES （1）

10. Do you feel dry in eyes？ NO （0） YES （1）

11. Do you have ocular inching？ NO （0） YES （1）

12. Do you have more secretion of the eyes？ NO （0） YES （1）

13. Do you often have tinnitus？ NO （0） YES （1）

14. Do you have dry pharynx？ NO （0） YES （1）

15. Do you have sore-throat？ NO （0） YES （1）

16. Do you have acne？ NO （0） YES （1）

17. Do you often feel dizzy？ NO （0） YES （1）

18. Do you have more scurf？ NO （0） YES （1）

19. Do you have dryness-heat？ NO （0） YES （1）

20. Do you have low-grade fever？ NO （0） YES （1）

21. Do you have insomnia？ NO （0） YES （1）

22. Do you have tantrum？ NO （0） YES （1）

23. Do you have yellow urine？ NO （0） YES （1）

24. Do you have constipation？ NO （0） YES （1）

25. Do you have red tongue？ NO （0） YES （1）

26. Do you have yellow musci？ NO （0） YES （1）

27. Do you have frequent and slim pulse？ NO （0） YES （1）

28. Do you have frequent and great pulse？ NO （0） YES （1）

Researcher’s name ： Site： Time：

Diagnosis result：
