# Supplementary material for: Quantization and diagnosis of Shanghuo (Heatiness) in Chinese medicine using a diagnostic scoring scheme and salivary biochemical parameters
Source: Chin Med. 2014 Jan 4;9:2. doi: 10.1186/1749-8546-9-2 (PMC3891990; doi:10.1186/1749-8546-9-2)
Supplement: Additional file 2 — The indexs and values of heatiness symptom. [file 1749-8546-9-2-S2.docx]

Value of Each Index

|  | Healthy Group（N=60） | | | | | | Patients with *Heatiness* (N=121) | | | | | |  |
| --- | --- | --- | --- | --- | --- | --- | --- | --- | --- | --- | --- | --- | --- |
| Indices | Negative（N） | P (%) | Negative Exponential Value | Positive（N） | P (%) | Positive Exponential Value | Negative（N） | P (%) | Negative Exponential Value | Positive（N） | P (%) | Positive Exponential Value | Value |
| (1) Ulcer | 60 | 100 | 10 | 0 | 0 | -10 | 39 | 32.2 | 5 | 82 | 67.8 | 8 | 23 |
| (2) Dry mouth | 50 | 83.3 | 9 | 10 | 16.7 | 2 | 20 | 16.5 | 2 | 101 | 83.5 | 9 | 14 |
| (3) Bitterness in the mouth | 59 | 98.3 | 10 | 1 | 1.7 | -8 | 84 | 69.4 | 8 | 37 | 30.6 | 5 | 15 |
| (4) Bad breath | 60 | 100 | 10 | 0 | 0 | -10 | 81 | 66.9 | 8 | 40 | 33.1 | 5 | 17 |
| (5) Gum swelling and aching or bleeding | 55 | 91.7 | 10 | 5 | 8.3 | -1 | 23 | 19 | 3 | 98 | 81 | 9 | 17 |
| (6) Nasal obstruction | 55 | 91.7 | 10 | 5 | 8.3 | -1 | 89 | 73.6 | 9 | 32 | 26.4 | 4 | 6 |
| (7) Nasal dryness | 58 | 96.7 | 10 | 2 | 3.3 | -5 | 57 | 47.1 | 7 | 64 | 52.9 | 7 | 15 |
| (8) Nose bleeding | 60 | 100 | 10 | 0 | 0 | -10 | 109 | 90.1 | 10 | 12 | 9.9 | 0 | 10 |
| (9) Xerophthalmus | 50 | 83.3 | 9 | 10 | 16.7 | 2 | 46 | 38 | 6 | 75 | 62 | 8 | 9 |
| (10) Eye itching | 57 | 95 | 10 | 3 | 5 | -3 | 87 | 71.9 | 9 | 34 | 28.1 | 4 | 8 |
| (11) Secretion of the eyes | 59 | 98.3 | 10 | 1 | 1.7 | -8 | 66 | 54.5 | 7 | 55 | 45.5 | 7 | 18 |
| (12) Tinnitus | 59 | 98.3 | 10 | 1 | 1.7 | -8 | 101 | 83.5 | 9 | 20 | 16.5 | 2 | 11 |
| (13) Pharyngoxerosis | 57 | 95 | 10 | 3 | 5 | -3 | 56 | 46.3 | 7 | 65 | 53.7 | 7 | 13 |
| (14) Sore throat | 59 | 98.3 | 10 | 1 | 1.7 | -8 | 83 | 68.6 | 8 | 38 | 31.4 | 5 | 15 |
| (15) Acne | 48 | 80 | 9 | 12 | 20 | 3 | 55 | 45.5 | 7 | 66 | 54.5 | 7 | 6 |
| (16) Dizziness | 55 | 91.7 | 10 | 5 | 8.3 | -1 | 90 | 74.4 | 9 | 31 | 25.6 | 4 | 6 |
| (17) Scurf desquamation | 55 | 91.7 | 10 | 5 | 8.3 | -1 | 89 | 73.6 | 9 | 32 | 26.4 | 4 | 6 |
| (18) Dryness-heat | 60 | 100 | 10 | 0 | 0 | -10 | 83 | 68.6 | 8 | 38 | 31.4 | 5 | 17 |
| (19) Low grade fever | 60 | 100 | 10 | 0 | 0 | -10 | 89 | 73.6 | 9 | 32 | 26.4 | 4 | 15 |
| (20) Insomnia | 53 | 88.3 | 9 | 7 | 11.7 | 1 | 57 | 47.1 | 7 | 64 | 52.9 | 7 | 8 |
| (21) Tantrum | 59 | 98.3 | 10 | 1 | 1.7 | -8 | 67 | 55.4 | 7 | 54 | 44.6 | 6 | 17 |
| (22) Yellow urine | 55 | 91.7 | 10 | 5 | 8.3 | -1 | 72 | 59.5 | 8 | 49 | 40.5 | 6 | 9 |
| (23) Constipation | 53 | 88.3 | 9 | 7 | 11.7 | 1 | 61 | 50.4 | 7 | 60 | 49.6 | 7 | 8 |
| (24) Red tongue | 58 | 96.7 | 10 | 2 | 3.3 | -5 | 97 | 80.2 | 9 | 24 | 19.8 | 3 | 9 |
| (25) Yellow coating | 59 | 98.3 | 10 | 1 | 1.7 | -8 | 83 | 68.6 | 8 | 38 | 31.4 | 5 | 15 |
| (26) Frequent and weak pulse | 55 | 91.7 | 10 | 5 | 8.3 | -1 | 62 | 51.2 | 7 | 59 | 48.8 | 7 | 11 |
| (27)Frequent and strong pulse | 60 | 100 | 10 | 0 | 0 | -10 | 104 | 86 | 9 | 17 | 14 | 2 | 13 |
